# Long-term effect of physical inactivity on osteosarcopenic obesity – a MRI-based investigation from a population-based cohort

**DOI:** 10.1186/s12891-025-09266-8

**Published:** 2025-10-28

**Authors:** Lena Sophie Kiefer, Roberto Lorbeer, Susanne Rospleszcz, Wolfgang Rathmann, Christa Meisinger, Annette Peters, Christopher L. Schlett, Fabian Bamberg, Sven S. Walter, Elke Maurer

**Affiliations:** 1 Department of Nuclear Medicine and Clinical Molecular Imaging, University Department of Radiology, University Hospital Tuebingen, Tuebingen, Germany; 2 Department of Radiology, Ludwig-Maximilian-University Hospital, Marchioninistraße 15, Munich, 81377 Germany; 3 Institute of Epidemiology, Helmholtz Zentrum Munich, German Research Center for Environmental Health, Neuherberg, Germany; 4 Medical Faculty, Institute for Medical Information Processing, Biometry and Epidemiology, Ludwig-Maximilians-University Munich, Munich, Germany; 5 German Center for Diabetes Research (DZD), Partner Düsseldorf, Munich-Neuherberg, Germany; 6 Institute for Biometrics and Epidemiology, German Diabetes Center, Duesseldorf, Germany; 7 Department of Diagnostic and Interventional Radiology, Medical Center, University of Freiburg, Freiburg, Germany; 8 Department for Diagnostic and Interventional Radiology, Eberhard Karls University Tuebingen, University Hospital Tuebingen, Tuebingen, 72076 Germany; 9 Department for Trauma and Reconstructive Surgery, BG Unfallklinik Tuebingen, Tuebingen, Germany; 10 LUMEDIS - Orthopäden, Kaiserstraße 14, Frankfurt, 60311 Germany

**Keywords:** Osteosarcopenic obesity, Population based Cohort Studies, Physical inactivity, Spine, Magnetic Resonance Imaging

## Abstract

**Background:**

The hazardous triad of osteopenia, sarcopenia and obesity was recently defined as osteosarcopenic obesity (OSO). The causes for OSO appear to be multifactorial, including age and gender, as well as chronic diseases. The impact of physical inactivity has not been studied so far.

**Purpose:**

The purpose of this study was to assess the association of short-term and long-term physical inactivity over a time period of 14 years on osteosarcopenic obesity in a population-based cohort from southern Germany.

**Methods:**

Supine whole body MRI (3 T scanner, Magnetom Skyra, Siemens Healthcare) was performed in 400 subjects from the population-based observational cohort study “Kooperative Gesundheitsforschung in der Region Augsburg (KORA)” to determine bone marrow fat fraction (BMFF), skeletal muscle fat fraction (SMFF) and total adipose tissue (TAT). Based on this, phenotyping was conducted into the groups of the OSO complex. Physical inactivity was obtained via a questionnaire at three timepoints: exam 1 (1999 to 2001), exam 2 (2006 to 2008) and exam 3 (2013 and 2014).

**Results:**

In total, 363 subjects (56.0 ± 9.1 years, 57.6% male) were included. The OSO phenotype was fully expressed in 81 (22,3%) participants. All pathological subgroups of the OSO complex, except isolated obesity were associated with less physical activity (< 1 h/week) at exam 3. Work activity correlated with the isolated osteopenic and sarcopenic phenotypes, as well as OSO, whereas neither walking, nor cycling activity correlated significantly with any phenotypic subgroup. Similarly, long-term physical inactivity was accompanied by isolated osteopenia, sarcopenia and osteosarcopenic obesity but not with isolated obesity. Lower back pain was present in 54.5% of all participants at exam 3. No correlation was shown with the OSO complex.

**Conclusion:**

Physical inactivity was strongly correlated with an isolated osteopenic, sarcopenic and OSO phenotype, but not with an isolated obese phenotype by MRI. Although slightly over fifty percent of participants reported back pain on exam 3, the manifestation of the OSO complex had no effect on this.

## Highlights

Short-term physical inactivity was associated with all subgroups of the Osteosarcopenic obesity (OSO) complex except isolated obesity.

Long-term physical inactivity was associated with isolated osteopenia, sarcopenia and osteosarcopenic obesity but not with isolated obesity.

There was no correlation between lower back pain and the OSO complex.

## Background context

Osteosarcopenic obesity (OSO), a condition characterized by the coexistence of decreased bone mineral density (osteoporosis/osteopenia), reduced muscle mass, with consecutively decreased strength and functional capacity (sarcopenia) and disproportionate increase in bodyweight (obesity), has increasingly gained attention lately [1–4]. Due to the heterogeneity of diagnostic techniques and the inconsistent definition of the term, it is hardly possible to determine the exact prevalence yet. In the current literature, prevalence rates vary between 4,7% to 14,5% if all three components of the OSO complex apply [1] and 0,7% to 7,2% in case of osteosarcopenia without comorbid obesity [1, 4, 5].

In this study, we used magnetic resonance imaging (MRI) due to its ability to acquire high-resolution images that enable detailed visualization of various soft tissue components, including muscle, fat, and water, based on their distinct molecular properties. Compared to commonly used methods such as dual-energy X-ray absorptiometry (DEXA) or bioelectrical impedance analysis (BIA), MRI offers simultaneous and compartment-specific quantification of skeletal muscle, visceral and subcutaneous adipose tissue, as well as ectopic lipid deposits. Additionally, MRI can assess bone characteristics, including fat infiltration in the bone marrow, which is relevant for capturing the osteopenic component of the osteosarcopenic obesity (OSO) phenotype [6, 7].

Although there was evidence already a decade ago that osteoporosis, sarcopenia, and obesity are closely related to each other, the triad as such remains poorly investigated [8]. OSO begins with either one or more of the three components and is usually progressive in nature. It is assumed that changes at a cellular level (e.g. decrease in osteoblasts) may lead to systemic changes (e.g. altered osteokines), which in turn present themselves as physical changes (e.g. reduced bone mass) and finally manifest clinically as osteoporosis, sarcopenia and/or obesity [9].

The causes therefore appear to be multifactorial. Risk factors common to the three components of OSO are age and sex [2, 10]. Evidence was obtained that with increasing age, the white abdominal fat also increases [11]. Likewise, the fat content of skeletal muscles and bones rises [12]. Further common risk factors are chronic stress, inflammation and inflammatory and/or endocrine diseases, as well as physical inactivity [5, 10, 13, 14]. Inadequate physical activity lacks a regular stimulus that helps maintain bone quality and mass [15]. On the other hand, a regular physical activity has a positive effect on muscle mass and strength [16, 17] by recruiting muscle satellite cells [18]. Additive endurance training promotes the release of growth factors, which in turn drives differentiation and proliferation [19], whilst strength training leads to an increase in the size (hypertrophy) and number (hyperplasia) of myofibrils [20].

Therefore, the aim of this study was to investigate associations of recent and long-term physical inactivity and the presence osteosarcopenic obesity (OSO) obtained in supine position by MRI.

## Material and methods

### Study design and population sample

Participants were derived from the “Cooperative Health Research in the Region of Augsburg” (KORA) S4-study (N = 4261) with a baseline examination in 1999 to 2001 (exam 1), a follow-up examination in 2006 to 2008 (exam 2) and a second follow-up in 2013 and 2014 (exam 3). Hereof, 400 participants received a whole-body MRI during exam 3 [21].

Participants were considered eligible for inclusion if they were willing to undergo a whole-body MRI and were classified into one of the following groups based on health assessment: prediabetes, diabetes, or control. Individuals were excluded from the study if they were older than 72 years, had a history (self-reported or confirmed) of stroke, myocardial infarction, or revascularization, or had contraindications for MRI such as a cardiac pacemaker, implantable defibrillator, cerebral aneurysm clip, neural stimulator, ear implants, ocular foreign bodies, or any other implanted device. Additional exclusion criteria included pregnancy or breastfeeding, claustrophobia, known allergies to gadolinium-based contrast agents, or a serum creatinine level of ≥ 1.3 mg/dL [22].

Approval was given by the institutional review board of the Ludwig Maximilian’s University Munich (Germany). Written consent was obtained from every participating subject.

### MR imaging protocol

During exam 3 in total 400 whole-body MR examinations were performed on a 3 Tesla scanner (Magnetom Skyra, Siemens Healthcare, Erlangen, Germany). A detailed description of the technical procedure as well as imaging protocols can be found elsewhere [22].

Briefly summarized, the musculoskeletal protocol embedded a dual-echo Dixon sequence (matrix: matrix: 256 × 256, field of view (FOV): 488 × 716 mm, echo time (TE) 1.26 ms and 2.49 ms, repetition time (TR): 4.06 ms, partition segments: 1.7 mm, flip angle: 9°) and a T2w single shot fast spin echo (SS-FSE) sequence (matrix: matrix: 320 × 200, field of view (FOV): 296 × 380 mm, echo time (TE) 91 ms, repetition time (TR): 1000 ms, partition segments: 5 mm, flip angle: 131°) [23].

A 2-point T1-weighted VIBE sequences (repetition time (TR): 4.06 ms; time to echo (TEs): 1.26 ms and 2.49 ms; flip angle 4°; slice thickness 1.7 mm) was used to determine bone marrow fat fraction (BMFF) in lumbar vertebrae L1 and L2 [24]. T2*-corrected, multi-echo 3D-gradient-echo Dixon-based sequence (repetition time (TR): 8.90 ms; TEs: 1.23 ms, 2.46 ms, 3.69 ms 4.92 ms, 6.15 ms and 7.38 ms; flip angle 4°, slice thickness 4 mm) was performed to measure skeletal muscle fat fraction (SMFF) in lumbar vertebrae L3 [25]. Based on a VIBE-Dixon sequence (TR: 4.06 ms; TEs: 1.26, 2.49 ms; flip angle 9°; slice thickness: 1.7 mm) VAT and SAT were quantified on a calculated fat-selective tomogram. VAT and SAT together form total adipose tissue (TAT) [22].

Body composition analysis was performed by determining MR imaging biomarkers of bone marrow fat fraction (BMFF), skeletal muscle fat fraction (SMFF) and visceral (VAT) and subcutaneous adipose tissue (SAT). Osteopenia and osteoporosis, characterized by reduced bone mineral density (BMD), have been described as "bone obesity," with recent data suggesting that increased bone marrow fat fraction (BMFF) inversely correlates with BMD, thus making BMFF a potential imaging biomarker for the osteopenic phenotype [7]. Phenotypic assignment to the OSO complex was performed based on these components.

### Outcome definition of osteosarcopenic obesity subgroups

The sex-specific median was calculated for the biomarkers of bone marrow fat fraction (BMFF), skeletal muscle fat fraction (SMFF) and total adipose tissue (TAT). A value greater than the median BMFF, SMFF or TAT has been classified as an osteopenic, sarcopenic or obese phenotype, whereas a value less-than-equal was classified as a healthy phenotype (Table 1).

**Table 1:** Phenotypic subgroups of osteosarcopenic obesity

	**Healthy**	**Isolated Osteopenia**	**Isolated Sarcopenia**	**Isolated Obesity**	**Osteopenic Sarcopenia**	**Osteopenic Obesity**	**Sarcopenic Obesity**	**Osteosarcopenic Obesity**
BMFF (Sex-specific median)	≤	>	≤	≤	>	>	≤	>
SMFF (Sex-specific median)	≤	≤	>	≤	>	≤	>	>
TAT (Sex-specific median)	≤	≤	≤	>	≤	>	>	>

### Risk factor measurements of physical inactivity

In our cohort, physical inactivity was measured via a standardized questionnaire at exam 1 (1999–2001), exam 2 (2006–2008) and exam 3 (2013–2014) using a single-choice question with a four graded answer as previously described [23].

#### Exercise inactivity per week

- No physical activity
- irregularly for ≤ 1 h
- regularly for ≥ 1 h
- regularly for ≥ 2 h

Referring to a previous study [23], this was used to calculate a *dichotomous variable* with.

- physical activity irregularly ≤ 1 h per week
- physical activity regularly ≥ 1 h per week.

Furthermore, two different longitudinal variables were generated: The first longitudinal variable was calculated of physical activity performed regularly ≥ 1 h over the time course of 14 years with.

- three-times (exam 1, exam 2 and exam 3)
- two-times (at two exams out of three)
- one-time (at one exam out of three)
- never (in any of the three exams).

The second longitudinal variable was gathered by summing up physical inactivity categories of all three exams with one point for physical activity regularly for ≥ 2 h, two points for physical activity regularly ≥ 1 h, 3 points for physical activity irregularly for ≤ 1 h and 4 points for no physical activity. This results in in values from 3 to 12; with a value of 3 indicating physical activity regularly performed for ≥ 2 h per week during all three examinations and a value of 12 representing no physical activity at any of the three exams [23].

Furthermore, information on work inactivity, as well as levels of inactivity related to walking and cycling, were collected through a four-level question for each category.

### Work inactivity

- no relevant physical labor
- light physical labor
- moderate physical labor
- heavy physical labor

### Walk inactivity (in minutes (min) per day)

- 15 min
- 15 to 30 min
- 30 to 60 min
- >60 min

### Cycling inactivity (in minutes per day)

- 15 min
- 15 to 30 min
- 30 to 60 min
- >60 min

### Lower back pain

Lower back pain was investigated via a single-choice question with a five graded answer:

- no back pain
- little back pain
- moderate back pain
- strong back pain
- very strong back pain

We chose a descriptive method to assess lower back pain using a single-choice question with five graded response options (ranging from "no" to "very strong" back pain) in order to capture not only pain intensity but also subjective differences in pain perception. This graded self-assessment allows for a practical and real-life relevant classification of the symptoms, particularly in chronic conditions such as osteosarcopenic obesity.

### Covariates

The division into healthy, prediabetic, and diabetic was determined on the basis of the oral glucose tolerance test (OGTT) and fasting glucose levels*: Impaired Fasting Glucose (IFG): FPG 5.6–6.9 mmol/L and Impaired Glucose Tolerance (IGT): OGTT 7.8–11.0 mmol/L. Subjects were categorized as having prediabetes if they met the criteria for IFG and/or IGT. However, if either the IFG or IGT criteria for diabetes mellitus were fulfilled (i.e., Diabetes Mellitus: FPG* ≥ *7.0 mmol/L and/or OGTT* ≥ *11.1 mmol/L and Healthy Controls: FPG* < *5.6 mmol/L and OGTT* < *7.8 mmol/L), the subject was classified as having diabetes mellitus. This approach ensured a clear and consistent classification based on standard diagnostic thresholds.*

BMI was calculated as the subjects’ weight in kilograms (kg)/height in meters squared (m^2^). A blood pressure ≥ 140/90 mmHg was defined as hypertension. Smoking status was recorded as a single choice question with three options: current smoker, former smoker, or never smoker. A detailed overview has been published elsewhere [26].

### Statistical analysis

Descriptive characteristics of participants during last examination are provided as means with standard deviations for continuous measurements and as absolute numbers and proportions for categorical measurements. Venn and bar diagrams were used to illustrated distribution of OSO subgroups. The correlations of OSO subgroups with proportion of irregular physical activity and back pain were evaluated by chi-square test, respectively.

Associations between physical inactivity and OSO were assessed by multinomial logistic regression models adjusted for age, sex, smoking, hypertension, and diabetes mellitus. Relative risk ratios (RRR) together with 95% confidence intervals (95% CI) were calculated. Physical active and healthy subjects were considered as reference group.

A *p*-value of < 0.05 was considered statistically significant. Statistical analyses were performed using Stata 16.1 (Stata Corporation, College Station, TX, U.S.A.).

## Results

**Fig. 1:**
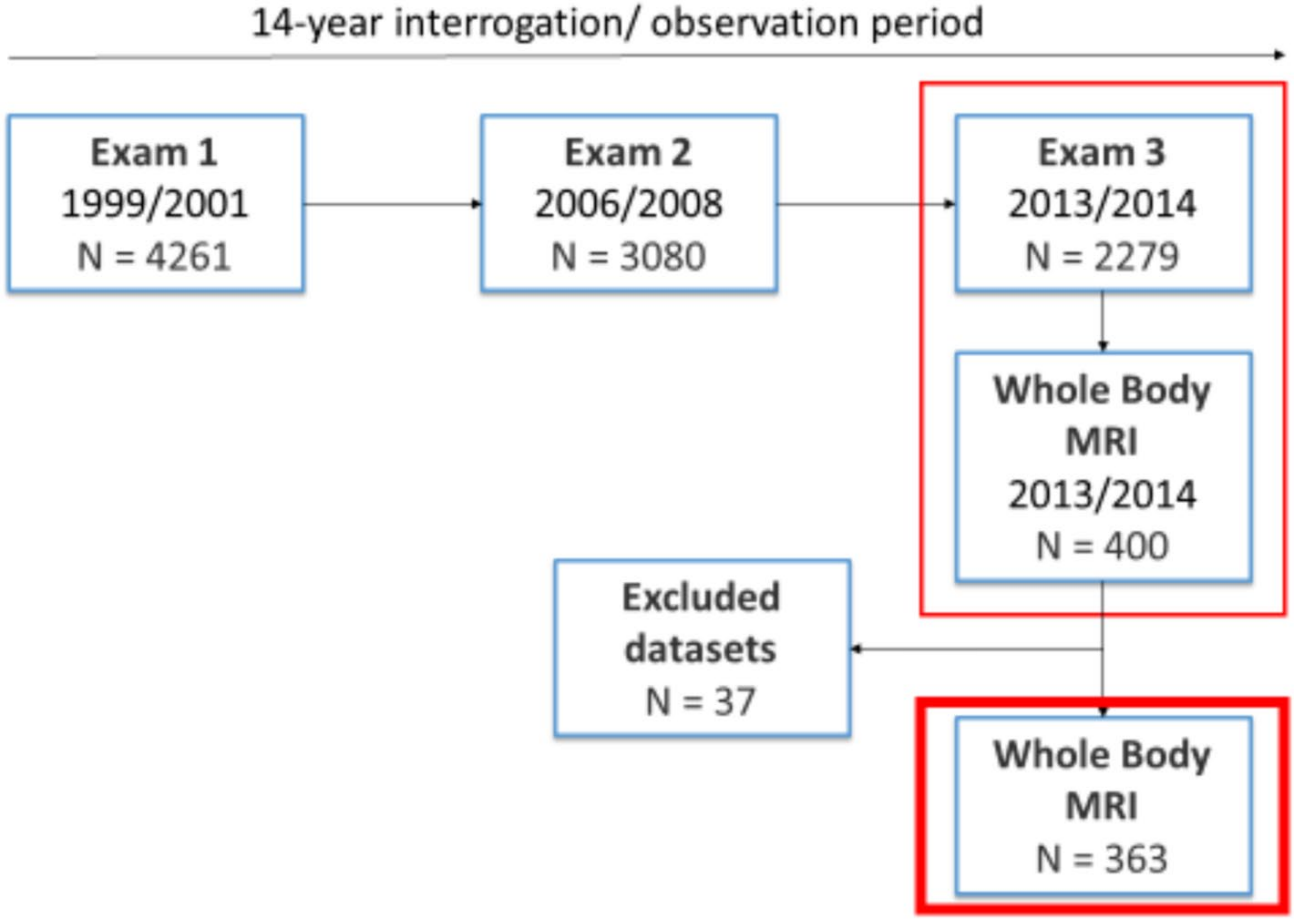
Participants’ flow chart

Out of the 400 participants included in this study, 37 were excluded due to poor image quality **(**Fig. 1**)**. The mean age of the included subjects was 56.0 ± 9.1 years with 57.6% being male. Detailed demographics and characteristics of the participants are provided in Table 2.

**Table 2:** Participants’ characteristics

Exam 3	All *N* = 363
Age (years)	56.0 (± 9.1)
Men	209 (57.6%)
Smoking status	
never smoker	133 (36.6%)
ex-smoker	156 (43%)
current smoker	74 (20.4%)
BMI (kg/m^2^)	27.9 (± 4.6)
Hypertension	118 (32.5%)
Known Diabetes mellitus	50 (13.8%)
Total Adipose Tissue (l)	12.5 (± 5.3)
Bone Marrow Fat Fraction (%)	54.3 (± 10.2)
Skeletal Muscle Fat Fraction (%)	12.3 (± 5.2)

Our analysis revealed that participants with increased adiposity, regardless of the specific phenotype, showed elevated fasting glucose and HbA1c levels, indicating reduced glycemic control compared to individuals without features of the osteosarcopenic adiposity (OSA) complex. Conversely, those in the "normal" group—without OSA characteristics—had the most favorable glucose profiles. Notably, the OSA subgroup demonstrated the highest rate of impaired glucose tolerance (63%), supporting a strong link between disturbances in glucose metabolism and distinct OSA phenotypes [7].

An overview of the distribution of participants based on their physical activity is presented in Table 3, showcasing the percentage breakdown.

**Table 3:** Distribution of participants based on their physical activity at exam 3

	**All *N* = 363**
Physical activity (Exam 3)	
≥ 1 h, regularly	218 (60.1%)
≤ 1 h, irregularly	145 (39.9%)
Work activity (Exam 3)	
heavy/moderate	104 (28.7%)
light/not relevant	259 (71.4%)
Walk activity (Exam 3)	
> 0.5 h daily	270 (74.4%)
< = 0.5 h	93 (25.6%)
Cycling activity (Exam 3)	
> 0.5 h daily	110 (30.3%)
< = 0.5 h	253 (69.7%)
Physical activity	
14 years ago (Exam 1) *N* = 362	
≥ 1 h, regularly	182 (50.3%)
≤ 1 h, irregularly	180 (49.7%)
7 years ago (Exam 2) *N* = 343	
≥ 1 h, regularly	199 (58.0%)
≤ 1 h, irregularly	144 (42.0%)
(≥ 1 h, regularly over 14 years) (Exam 1 – Exam 3) *N* = 343	
three/two times	188 (54.8%)
one times/never	155 (45.2%)

**Fig. 2:**
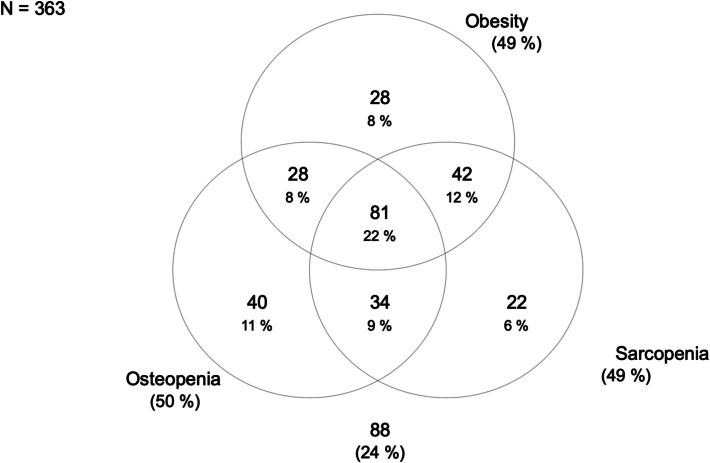
Subject allocation to the phenotypic subgroups of osteosarcopenic obesity

In total, 88 (24.2%) were classified with a healthy phenotype by MRI. An isolated osteopenic phenotype was found in 40 (11,0%) subjects, an isolated sarcopenic phenotype in 22 (6,1%) and an isolated obese phenotype in 28 (7,7%). 81 (22,3%) participants were allocated to an osteosarcopenic obese phenotype by MRI (Fig. 2).

### Physical inactivity

**Fig. 3:**
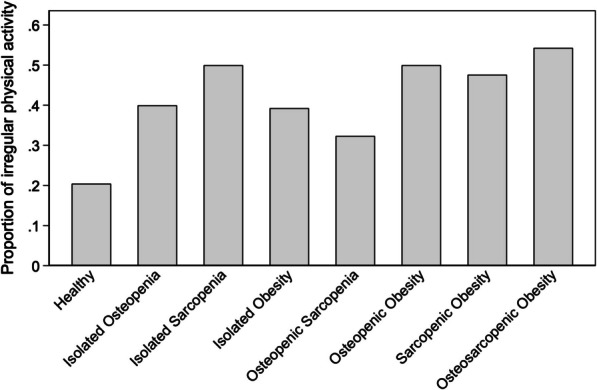
Proportions of irregular physical activity depending on the subgroups of the OSO complex (p = 0.001)

The proportion of irregular physical activity varies significantly between the subgroups of the OSO complex. While the share was lowest in the group of subjects with a healthy phenotype, the percentage was highest in the group with complete pattern of OSO phenotype. Notably, the subgroups with an sarcopenic phenotype showed a higher proportion of irregular physical activity compared to other subtypes (Fig. 3).

Physical activity less than one hour per week at exam 3 was significantly associated with all pathological subgroups of the OSO complex, except isolated obesity, in which the association was scarcely not significant. Instead, work activity only impacted on isolated osteopenia and sarcopenia, as well as on sarcopenic obesity and osteosarcopenic obesity. Neither walking, nor cycling activity were associated with any phenotypic subgroup of the OSO complex (Table 4).

**Table 4:** Association between current physical activity (exam 3) and osteosarcopenic obesity

	**Isolated Osteopenia** **RRR (95%CI)**	**p**	**Isolated Sarcopenia** **RRR (95%CI)**	**p**	**Isolated Obesity** **RRR (95%CI)**	**p**	**Osteopenic Sarcopenia** **RRR (95%CI)**	**p**	**Osteopenic Obesity** **RRR (95%CI)**	**p**	**Sarcopenic Obesity** **RRR (95%CI)**	**p**	**Osteosarcopenic Obesity** **RRR (95%CI)**	**p**
**Physical activity**														
≥ *1 h, regularly*	Ref		Ref		Ref		Ref		Ref		Ref		Ref	
≤ *1 h, irregularly*	3.59 (1.48; 8.70)	**0.005**	6.32 (2.13; 18.77)	**0.001**	2.42 (0.92; 6.37)	0.074	3.33 (1.2; 9.24)	**0.021**	4.09 (1.51; 11.1)	**0.006**	4.61 (1.89; 11.21)	**0.001**	6.87 (2.94; 16.04)	** < 0.001**
**Work activity**														
* heavy/moderate*	Ref		Ref		Ref		Ref		Ref		Ref		Ref	
* light/not relevant*	0.36 (0.15; 0.87)	**0.024**	0.31 (0.1; 0.96)	**0.043**	0.9 (0.31; 2.63)	0.851	0.56 (0.18; 1.74)	0.314	0.55 (0.19; 1.6)	0.272	0.27 (0.11; 0.67)	**0.004**	0.23 (0.1; 0.55)	**0.001**
**Walk activity**														
> *0.5 h daily*	Ref		Ref		Ref		Ref		Ref		Ref		Ref	
< = *0.5 h*	0.94 (0.4; 2.2)	0.885	0.96 (0.31; 2.95)	0.949	1.01 (0.41; 2.51)	0.978	0.55 (0.18; 1.65)	0.289	0.33 (0.1; 1.09)	0.068	0.58 (0.23; 1.46)	0.25	0.63 (0.27; 1.48)	0.286
**Cycling activity**														
> *0.5 h daily*	Ref		Ref		Ref		Ref		Ref		Ref		Ref	
< = *0.5 h*	1.69 (0.69; 4.16)	0.251	1.58 (0.52; 4.77)	0.42	1.38 (0.5; 3.8)	0.534	1.36 (0.52; 3.57)	0.528	1.12 (0.42; 2.97)	0.821	1.90 (0.77; 4.69)	0.166	2.06 (0.91; 4.65)	0.083

Relative risk ratios (RRR) are from multinomial logistic regression models (with the healthy group as reference) adjusted for age, sex, smoking, hypertension, and diabetes mellitus

Long-term physical inactivity was substantially associated with isolated osteopenia and sarcopenia but not with isolated obesity. Osteopenic obesity and osteosarcopenic obesity were impacted by physical inactivity over a time period of 14 years (Table 5).

**Table 5:** Association between longitudinally measured physical activity (exam 1, exam 2 and exam 3) and osteosarcopenic obesity

Physical activity	Isolated Osteopenia RRR (95%CI)	p	Isolated Sarcopenia RRR (95%CI)	p	Isolated Obesity RRR (95%CI)	p	Osteopenic Sarcopenia RRR (95%CI)	p	Osteopenic Obesity RRR (95%CI)	p	Sarcopenic Obesity RRR (95%CI)	p	Osteosarcopenic Obesity RRR (95%CI)	p
**14 years ago** (n = 362)														
≥ *1 h, regularly*	Ref		Ref		Ref		Ref		Ref		Ref		Ref	
≤ *1 h, irregularly*	2.05 (0.91; 4.62)	0.082	2.88 (1.01; 8.16)	**0.047**	0.98 (0.39; 2.42)	0.957	1.37 (0.53; 3.55)	0.516	5.80 (2.02; 16.71)	**0.001**	1.54 (0.68; 3.47)	0.304	2.1 (0.98; 4.51)	0.057
**7 years ago** (*n* = 343)														
≥ *1 h, regularly*	Ref		Ref		Ref		Ref		Ref		Ref		Ref	
≤ *1 h, irregularly*	2.35 (1.02; 5.45)	**0.046**	2.69 (0.93; 7.81)	0.068	1.13 (0.44; 2.88)	0.797	1.48 (0.56; 3.92)	0.434	3.17 (1.22; 8.25)	**0.018**	1.11 (0.46; 2.67)	0.822	2.12 (0.96; 4.68)	0.062
**(≥ 1 h, regularly over 14 years)** (*n* = 343)														
* three/two times*	Ref		Ref		Ref		Ref		Ref		Ref		Ref	
* one times/never*	2.8 (1.19; 6.6)	**0.018**	3.91 (1.32; 11.57)	**0.014**	1.55 (0.61; 3.97)	0.36	1.57 (0.57; 4.3)	0.384	7.29 (2.54; 20.9)	** < 0.001**	1.67 (0.69; 4.04)	0.256	3.6 (1.6; 8.11)	**0.002**
**summed over 14 years** (*n* = 343)	1.34 (1.14; 1.58)	** < 0.001**	1.46 (1.2; 1.79)	** < 0.001**	1.15 (0.96; 1.37)	0.133	1.22 (1.02; 1.46)	**0.028**	1.51 (1.25; 1.83)	** < 0.001**	1.25 (1.07; 1.48)	**0.006**	1.38 (1.18; 1.61)	** < 0.001**

Relative risk ratios (RRR) are from multinomial logistic regression models (with the healthy group as reference) adjusted for age, sex, smoking, hypertension, and diabetes mellitus

### Correlation between osteosarcopenic obesity and back pain (exam 3)

During the second follow-up, lower back pain was reported by 54.5% of all subjects. Among the participants, approximately one-third reported experiencing mild back pain, while 16.3% reported average back pain. 3,9% reported strong and 1,1% very strong back pain. Interestingly, there was no correlation between any phenotypic subgroup of the OSO complex and lower back pain (Table 6).

**Table 6:** Correlation between osteosarcopenic obesity and back pain (exam 3). Data is given in n (%)

	**All** ***N* = 363**	**Healthy** ***N* = 88**	**Isolated Osteopenia** ***N* = 40**	**Isolated Sarcopenia** ***N* = 22**	**Isolated Obesity** ***N* = 28**	**Osteopenic Sarcopenia** ***N* = 34**	**Osteopenic Obesity** ***N* = 28**	**Sarcopenic Obesity** ***N* = 42**	**Osteosarcopenic Obesity** ***N* = 81**
**Back pain, n (%)**									
*Not at all*	165 (45.5%)	42 (47.7%)	22 (55%)	10 (45.5%)	12 (42.9%)	14 (41.2%)	11 (39.3%)	16 (38.1%)	38 (46.9%)
*Little*	121 (33.3%)	29 (33%)	12 (30%)	8 (36.4%)	9 (32.1%)	13 (38.2%)	13 (46.4%)	15 (35.7%)	22 (27.2%)
*Average*	59 (16.3%)	15 (17.1%)	5 (12.5%)	4 (18.2%)	6 (21.4%)	7 (20.6%)	1 (3.6%)	10 (23.8%)	11 (13.6%)
*Strong*	14 (3.9%)	2 (2.3%)	1 (2.5%)	0 (0%)	0 (0%)	0 (0%)	3 (10.7%)	1 (2.4%)	7 (8.6%)
*Very strong*	4 (1.1%)	0 (0%)	0 (0%)	0 (0%)	1 (3.6%)	0 (0%)	0 (0%)	0 (0%)	3 (3.7%)

chi2-test, p = 0.258

## Discussion

The hazardous composite of osteopenia, sarcopenia and obesity was recently described as the OSO complex [1, 2, 14]. Although there was evidence of the close linkage of the three components a decade ago, little research has been done on the OSO complex as such [8]. Physical inactivity is among the risk factors considered [27].

Therefore, we aim to determine correlations between long-term physical inactivity and osteosarcopenic obesity (OSO) obtained in supine position by MRI.

Our results demonstrate, that physical inactivity was observed to be most widespread among patients with full expression of the OSO complex and less in healthy individuals. Predominantly, the proportion of inactive subjects was highest in the sarcopenic phenotype subgroup and in groups which included the sarcopenic phenotype. Physical inactivity during the second follow-up examination affected all manifestations of the OSO complex substantially. Activity related to work, was associated with an isolated osteopenic and sarcopenic phenotype, but not with ab isolated obese phenotype. In contrast, walking and cycling activity was not associated with any subgroup of the OSO complex. Long-term physical inactivity impacted significantly on the isolated osteopenic and sarcopenic phenotypes, osteopenic obese and osteosarcopenic obese phenotypes. There was no correlation between the OSO complex by MRI and lower back pain.

### Physical inactivity

Osteopenia/osteoporosis is characterized by the decrease in bone mass and a deterioration of the microarchitecture [28]. Besides increasing age, female sex is often considered to be a risk factor for osteoporosis [29]. Kirk et al. outline a close link between bone and muscle, proofing that age, sex and physical inactivity also have a negative impact on osteosarcopenia [10]. In line, we were able to show that short- and long-term physical inactivity is negatively associated with an osteopenic phenotype. But also over a period of 14 years, the detrimental association with osteosarcopenia could be demonstrated. Conversely, a physical load sets impulses in terms of stretching, traction or compression, which are recognized as stimuli by osteocytes. Repetitive exposure to these stimuli eventually leads to the activation of a cascade that promotes bone formation [30]. Thus, is not surprising, that physical activity has a protective effect on bone health.

Aging skeletal muscle is noticeable by a decrease in muscle mass and therefore muscle strength, known as sarcopenia [31]. This might be due to an age-related apoptosis of myocytes, damage of the macroscopic muscular structure by oxidative stress, an activation of pro-inflammatory cytokines and an oversupply of myostatin, which in turn negatively affects muscle growth through protein inhibition [32, 33]. In addition to primary aging, secondary aging, triggered by environmental factors and diseases, negatively impacts muscle degeneration. Thus, physical inactivity is considered to be a major determinant [31, 34]. Still, literature is controversial regarding the influence of physical inactivity on sarcopenia. On the one hand side, a sedentary lifestyle might promote sarcopenia by secondary aging [33]. We found physical inactivity at exam 3 and long-term inactivity associated with sarcopenic phenotype. This is in line with other studies [35, 36]. Conversely, regular physical activity may prevent sarcopenia [37], thus should be promoted more intensively in society [38]. On the other hand, Wu et al*.* stated, that after adjustment for sex and age, the impact of physical inactivity on sarcopenia was not significant, despite positive correlation [39].

Interestingly, factors responsible for secondary muscle aging might also affect other body compartments, causing obesity among others, and present as sarcopenic obesity [31]. A close inter-correlation between body fat content and sarcopenia was also shown by Tyrovolas et al*.* [37], with an increase in prevalence with age [40]. Our data demonstrate a strong association of the sarcopenic obese phenotype and physical inactivity at exam 3, however, the long-term impact seemed to be rather low. This is in line with other studies, proofing an association of less physical activity with sarcopenic obesity [37].

The causes of obesity are multifactorial. Thus, in addition to a genetic predisposition, physical inactivity combined with an increased/unbalanced food intake plays a decisive role [35]. The isolated obese phenotype was not associated with physical inactivity in our cohort. A decrease in prevalence was shown with age. However, the decrease was more likely due to an onset of sarcopenia and thus the shift to the sarcopenic obese phenotype than to physical weight loss [40]. Furthermore, an increase in adipose tissue is assumed to be another promotor of sarcopenia [41]. This allows to show the close linking of sarcopenia with obesity.

There is proof that physical activity prevents functional disability and increases functional capacity in osteosarcopenic obese women. The decisive factor, however, is that the physical activity is carried out in leisure time, but preferably under constant guidance, as in a gym [27]. Our results are in part contrary to these, as in addition to a benefit of regular leisure time physical activity of more than 1 h per week over a longer period of time, we were also able to show a positive effect from physically demanding professional work. Endurance-enhancing exercise, such as cycling or walking, were not associated with sarcopenic phenotypes. Some studies have shown that, however, through strength training muscle mass increases [35, 42]. But also vice versa, osteopenia, sarcopenia and obesity lead to an increase in frailty and decrease in physical function, which in turn increases morbidity and mortality [43]. Therefore, we consider it essential to examine further risk factors of OSO and sensitize the public for early detection.

To our knowledge, this is the first study analyzing the association of the OSO complex and back pain. In our cohort, there was no correlation between any subgroup of the OSO complex and lower back pain. Contrary to our results, one study found that osteopenic sarcopenia was associated with low back pain, while patients with osteoporosis alone failed to show a correlation [44]. Similarly, it was shown that lower back pain was associated with reduced muscle mass [45]. Still, further research is needed.

In terms of limitation, we only performed one single whole-body MRI per subject as part of exam 3. Thus, there are no former MR images to compare to. Second, neither functional measurement of skeletal muscle performance, nor clinically criteria were used to diagnose sarcopenia. Instead only supine MR imaging biomarkers for body composition phenotyping have been assessed.

Despite these limitations, the findings of our study remain robust and valuable. Whole-body MRI provides high-resolution, objective, and reproducible measurements of tissue composition, allowing for precise differentiation between muscle, adipose tissue, and bone marrow fat. These imaging biomarkers are increasingly recognized as reliable surrogates for musculoskeletal health and are particularly advantageous in large-scale, non-invasive population studies. Furthermore, our ability to demonstrate significant associations between long-term physical inactivity and distinct OSO phenotypes underlines the relevance and strength of the imaging data. Also, a differentiation of physical activity into recreational and competitive sports was not performed. Neither was made a discrimination between endurance, strength and precision sports.

Furthermore, other comorbidities and medication use were not the focus of the present analysis, as it specifically aimed to investigate osteosarcopenic adiposity (OSA) in relation to physical inactivity and lower back pain. However, their potential relevance is acknowledged, and related aspects—particularly cardiovascular risk factors—have been addressed in a previous publication by our group.

## Conclusion

Physical inactivity was strongly correlated with isolated osteopenia, sarcopenia and osteosarcopenic obesity but not with isolated obesity. Although slightly over fifty percent of participants reported back pain on exam 3, the manifestation of the OSO complex had no effect on this.

## Data Availability

The datasets used and/or analyzed during the study are available from the corresponding author on reasonable request.
